# Natural resource dependence, public education investment, and human capital accumulation

**DOI:** 10.1007/s12182-018-0235-0

**Published:** 2018-06-20

**Authors:** Hua-Ping Sun, Wei-Feng Sun, Yong Geng, Yu-Sheng Kong

**Affiliations:** 1 Institute of Industrial Economics, Jiangsu University, Zhenjiang, 212013 China; 2 School of Environmental Science and Engineering, Shanghai Jiao Tong University, Shanghai, 200240 China; 3 Department of Economics and Management, Yuncheng University, Yuncheng, 044000 China

**Keywords:** Natural resource dependence, Public education investment, Human capital accumulation, Resource curse

## Abstract

This paper examines the relationships between natural resource dependence, public education investment, and human capital accumulation. It addresses why the “blessing” of abundant natural resources often turns into a “curse” in many countries and regions, focusing on the crowding-out effect of natural resources on human capital. According to our empirical analysis of provincial panel data from China, natural resource dependence is significantly and negatively correlated with human capital accumulation. The crowding-out effect of natural resources on human capital exists only in the central and western regions of China. Our introduction of an interaction term for natural resource dependence and public education investment underscores the possibility of investing in public education to reduce the crowding-out effect of natural resource dependence on human capital. The government should utilize the income of the natural resource sector to increase investment in education to enhance local human capital.

## Introduction

Natural resources constitute an important material basis for national economic development and social progress. Indeed, abundant natural resources play an important role in the development of cities as well as in the wealth of a country. The industrial revolution was able to occur in the UK due to the rich coal and iron resources in its northern region. The long-term, rapid, and stable economic development of the USA, Canada, Germany, and the Nordic countries has also benefited from the rich natural resources in these countries. However, since the 1980s, a number of countries and regions with abundant natural resources have encountered development difficulties, and their economic growth rates have been significantly lower than those of countries with scarce resources. The average national incomes of most of the African countries with abundant resources and of most of the oil-rich Middle East countries are lower than the world average, as these countries have shown weak economic growth. The economies of countries and regions with poor resources, such as Japan, Singapore, South Korea, Hong Kong, differ significantly from those of the resource-rich countries, and the per capita income of these economies is high, as they show rapid and stable growth. The growth rate of many economies is negatively related to their resource abundance, a phenomenon termed the “resource curse” by Auty (1994).

Since Auty (1994), the question of whether natural resources are a “blessing” or a “curse” for economic development has become the focus of considerable debate. Although economists have produced a substantial theoretical and empirical literature on relevant issues, they have not yet reached a consensus. Based on a review of the literature related to the resource curse at home and abroad, Zhang et al. (2016) noted that, despite the doubts of some researchers and the ability of some countries and regions to avoid it, most countries and regions with abundant resources have, in fact, experienced the “resource curse”. However, it should be noted that the negative correlation between natural resources and economic growth is based on endogenous variables. Indeed, abundant resources are not the main reason for slow economic growth, and the most pressing unanswered question concerns why a resource blessing turns into a resource curse in many countries and regions.

The resource curse is a common phenomenon, but some countries and regions show that it can be avoided. Given that governments can adopt measures that prevent its occurrence, it is important to explore the causes of the resource curse and determine the mechanism by which it affects economic growth. Specifically, the crowding-out effect of natural resources on human capital is among the most important causes of the resource curse.

Human capital is the capacity of the population to drive economic development. A larger stock of human capital facilitates technological innovation. Education is the main contributor to human capital accumulation, so in this paper the accumulation of human capital is measured by the number of college students divided by the total population. The crowding-out effect of natural resources on human capital means the negative relationship between natural resource dependence and human capital accumulation. In most cases, the natural resource sector is a low-demand sector for human capital, so the development of the natural resource sector leads to an actual decline in the use of human capital in the whole economy. And the deepening of dependence on natural resources leads to a reduction in the investment demand for human capital.

This paper makes two contributions to the literature. First, although the effect of natural resource dependence on human capital accumulation has been extensively explored, it remains unknown whether this effect is positive or negative. We perform an empirical analysis based on panel data collected from 1999 to 2015 from 31 Chinese provinces. The results show that natural resource dependence has a significant negative impact on human capital. Second, although some recent studies also reported a crowding-out effect of natural resource dependence on human capital, they do not explore how to reduce or avoid this effect. To this end, we analyze the data from the perspective of investing in public education. Although many factors affect the accumulation of human capital, we focus on public education investment because this topic offers two obvious advantages. First, public education investment is the main contributor to the accumulation of human capital. Second, public education investment is under the direct control of the government. Therefore, an analysis of the impact of public education investment on the relationship between natural resource dependence and human capital accumulation may contribute to the development of public policy.

## Literature review

Economists advance two different views of the role of natural resources in the economy. The first holds that natural resources have a positive impact on economic growth, reflecting the resource blessing effect. The second holds that natural resources have a negative effect on economic growth, reflecting the resource curse effect. The resource blessing perspective can be traced back to Adam Smith (1776), and this optimistic view was widely accepted until the early 1980s. Gelb (1988) found that, from 1971 to 1983, oil producing economies suffered more efficiency losses with regard to capital formation than did non-oil-producing economies. As noted above, the term “resource curse” was first proposed by Auty (1994), who claims that countries with abundant natural resources are less able to utilize these resources to promote economic growth than are those countries with scarce resources, further stating that the former countries have lower growth rates. Based on these findings, Sachs and Warner (1997, 1999, 2001) conducted a series of empirical studies and reported that natural resource dependence is negatively correlated with economic growth.

The resource curse has attracted considerable academic attention in the twenty-first century. Scholars have used cross-sectional and panel data to re-examine the resource curse on both national and regional levels. Gilberthorpe and Papyrakis (2015) examined the current debates and evolution of the literature across disciplinary lines and the micro-, meso- and macrolevels on the resource curse. Papyrakis and Gerlagh (2007), Freeman (2009), and James and Aadland (2011) reported that the resource curse phenomenon exists at the state level in the USA. Badeeb et al. (2016) empirically examined the existence of an oil curse in the finance–growth nexus in Malaysia from the role of investment. They found that oil rent has an indirect impact on the finance–growth nexus through the quantitative channel or investment quantity. The policy implications of their findings are that policymakers should reduce dependence on oil and promote economic diversification. Similar empirical studies have been performed at the regional level in China. Xu and Wang (2006), Hu and Xiao (2007), Shao and Qi (2008), Shao et al. (2013), and Zhou and Guo (2015) reported the effect of the “resource curse” phenomenon at the regional level in China.

However, hypotheses about the resource curse face three main criticisms. First, the rationality of the natural resources metrics selection framework is the most vulnerable aspect of this formulation, as the use of different metrics inevitably produces different conclusions. Badeeb et al. (2017) reviewed the mechanisms and the empirical studies of the natural resource curse or on factors associated with economic growth. They found that the resource curse reflects empirical misspecification. Using the per capita approach to the measurement of the abundance of natural resources, Fang et al. (2011) argued that the resource curse hypothesis does not hold for the city level in China. Second, the endogenous problem of the explanatory variable is not easily controlled, and this is related to the measurement of natural resources. Indeed, as resource dependence is, to a certain extent, endogenous in relation to economic growth and institutional factors, it should not be treated as an exogenous explanatory variable in a regression model. Brunnschweiler (2008) controlled for the endogenous problem and reported that the results do not support the resource curse hypothesis. Third, the success of some countries with abundant natural resources also argues against this hypothesis. Specifically, successful cases in Chile, Norway, Malaysia, Botswana and other countries demonstrate that the resource curse is not a universal phenomenon.

At the same time, slow economic growth, “anti-industrialization” sentiments, income inequality, and corruption affect most countries/regions with abundant resources (Zhang et al. 2016). However, these challenges also show that the resource curse is not inevitable. Indeed, the resource curse emerges only under certain conditions. Therefore, it is important to determine the circumstances under which, and the mechanisms by which, the resource curse emerges, to develop policies to avoid and manage this phenomenon. Previous studies have focused primarily on the “Dutch disease”, the crowding-out effect of natural resources and the volatility of prices and systems. This paper, however examines the resource curse in terms of resource dependence and human capital.

Some researchers report that countries and regions with abundant resources lack human capital and believe that the crowding-out effect of natural resources on human capital is an important mechanism underpinning the resource curse. Gylfason (2001) and Birdsall et al. (2000) indicated that abundant resources are negatively related to the level of human capital and believed that the abundant resources decrease the investment of human capital, thereby inhibiting economic growth. Douangngeune et al. (2005) confirmed the crowding-out effect of land resources by comparing the educational level and economic development of Thailand, Japan, and South Korea. Auty (2007) and Han and Zhang (2015) reported that natural resource dependence has an inhibitory effect on human capital, whereas Blanco and Grier (2012) reported that overall resource dependence has no direct effect on human capital. However, after disaggregating the natural resource variable into sub-categories, they concluded that oil export dependence and agricultural export dependence have long-term negative effects on human capital.

Some studies concluded that the crowding-out effect of resource endowment on human capital is not inevitable, as some countries that have successfully avoided the resource curse have higher levels of human capital. Bravo-Ortega and Gregorio (2005) claimed that high levels of human capital can reduce the negative effect of natural resource dependence on the rate of economic growth. The empirical research performed by Stijns (2006) indicated that resource abundance is positively correlated with human capital. Moreover, the previously reported negative correlation between resource wealth and human capital is not robust. Stijns believed that the rational allocation of resource income is a prerequisite for the promotion of human capital accumulation in countries with abundant resources. Dahlman et al. (2006) reported that improving human capital by increasing equality and quality education shifted Finland’s economy from a resource-driven mode to a knowledge-driven mode.

Most studies on the relationship between natural resource dependence and human capital accumulation are empirical, and their conclusions are inconsistent. However, the mechanism that, theoretically, underpins the influence of natural resource dependence on human capital accumulation still needs to be explored. Jung and Thorbecke (2003) discussed the impact of public education expenditure on human capital, growth, and poverty based on a general equilibrium approach taking Tanzania and Zambia as examples. Blankenau and Simpson (2004) analyzed the relationship of public education expenditure and economic growth. Teles and Andrade (2004) discussed the role of public investment in basic education and economic growth. Birdsall et al. (2000) provided a conceptual framework for analyzing the effect of natural resource dependence on human capital. Shao and Yang (2014) outlined a mathematical model for analyzing the potential impact of resource dependence on human capital and economic growth. Dissou et al. (2016) analyzed the relationship of government spending on education, human capital accumulation, and growth. Using panel data of China’s 31 provinces, this paper theoretically and empirically investigates the effect of natural resource dependence on human capital. Public education investment, an area with clear policy implications, is treated as an intermediate variable in the relationship between natural resource dependence and human capital.

## Theoretical analysis

Economic growth depends on physical capital, human capital, technology, and other factors. In modern economies, human capital and technology are regarded as the engines of long-term economic growth. However, the crowding-out effect of resource dependence on human capital inevitably leads to slow economic growth in the long term and a low level of economic development. Here, we analyze the crowding-out effect of resource dependence on human capital.

This paper assumes that an economy has two sectors: the natural resource sector and the manufacturing sector. The demand for human capital in the natural resource sector is lower than in the manufacturing sector.

### Natural resource sector

The natural resource sector uses natural resources, physical capital, and human capital as inputs; it relies on the Cobb–Douglas production function and exhibits constant returns to scale. The production function can be expressed as follows:

1 $$Q_{\text{N}} = F_{\text{N}} (N,K_{\text{N}} ,H_{\text{N}} ) = A_{\text{N}} N^{\alpha } K_{\text{N}}^{\beta } H_{\text{N}}^{(1 - \alpha - \beta )}$$

where $N$ is the stock of natural resources; $K_{\text{N}}$ is the physical capital stock of the natural resource sector; $H_{\text{N}}$ is the human capital stock of the natural resource sector and can be expressed as the product of the average human capital level $h_{\text{N}}$ and employment $L_{\text{N}}$ in the natural resource sector; and $A_{\text{N}}$ represents the technical level of the natural resource sector and is assumed to be a constant. The development of the natural resource sector is influenced by the price of resource products much more than manufacturing products. If the price standard of a manufacturing product were 1 and the price of a natural resource product were $P_{\text{N}}$, then the production function of the natural resource sector could be further expressed as:

2 $$Q_{\text{N}} = P_{\text{N}} F_{\text{N}} (N,K_{\text{N}} ,H_{\text{N}} ) = P_{\text{N}} A_{\text{N}} N^{\alpha } K_{\text{N}}^{\beta } H_{\text{N}}^{(1 - \alpha - \beta )}$$

Thus, in the natural resource sector, the returns on natural resources are expressed as $R_{\text{N}} = \alpha P_{\text{N}} A_{\text{N}} N^{\alpha } K_{\text{N}}^{\beta } H_{\text{N}}^{(1 - \alpha - \beta )}$, and the returns on physical capital are expressed as $R_{{K_{\text{N}} }} = \beta P_{\text{N}} A_{\text{N}} N^{\alpha } K_{\text{N}}^{\beta } H_{\text{N}}^{(1 - \alpha - \beta )}$. The income of labor should include the income of labor input and the income of human capital. It is assumed that the two parts of income belong to labor. Thus, the labor income of the natural resource sector is $W_{{H_{\text{N}} }} = \left( {1 - \alpha - \beta } \right)P_{\text{N}} A_{\text{N}} N^{\alpha } K_{\text{N}}^{\beta } H_{\text{N}}^{(1 - \alpha - \beta )}$.

### Manufacturing sector

The development of manufacturing, which involves a higher demand for technological progress and human capital than the natural resource sector, is a sign of industrialization. Indeed, manufacturing requires the input of physical capital and human capital. The production function of the manufacturing sector is assumed to be the Cobb–Douglas-type production function with constant returns to scale:

3 $$Q_{\text{M}} = F_{\text{M}} \left( {K_{\text{M}} ,H_{\text{M}} } \right) = A_{\text{M}} K_{\text{M}}^{\gamma } H_{\text{M}}^{1 - \gamma }$$

where $K_{\text{M}}$ denotes the capital stock of the manufacturing sector; $H_{\text{M}}$ denotes the human capital stock of the manufacturing sector and can be expressed as the product of the average human capital level $h_{\text{M}}$ and employment $L_{\text{M}}$ in the manufacturing industry; and $A_{\text{M}}$ denotes the technical level of the manufacturing sector. The return of the manufacturing sector to material capital is $R_{{K_{\text{M}} }} = \gamma A_{\text{M}} K_{\text{M}}^{\gamma } H_{\text{M}}^{1 - \gamma }$, and the labor income of the manufacturing sector is $W_{{H_{\text{M}} }} = \left( {1 - \gamma } \right)A_{\text{M}} K_{\text{M}}^{\gamma } H_{\text{M}}^{1 - \gamma }$.

### Aggregate production function

The economy is divided into the natural resource sector and the manufacturing sector. The production function of the economy can be expressed as follows:

4 $$Q = F(N,K,H) = AN^{\lambda } K^{\theta } H^{(1 - \lambda - \theta )}$$

The economy is a function of constant returns to natural resources, physical capital, and human capital. $Q_{{}}$ denotes total output, $Q = Q_{\text{N}} + Q_{\text{M}}$. $K$ denotes physical capital investment, and $K = K_{\text{N}} + K_{\text{M}}$. Human capital $H$ can be expressed as the product of the average level of human capital $h$ and employee $L$, and $L = L_{\text{N}} + L_{\text{M}}$. ${\text{A}}$ denotes the general technical level. In this economy, we can use ${{Q_{\text{N}} } \mathord{\left/ {\vphantom {{Q_{\text{N}} } Q}} \right. \kern-0pt} Q}$ or ${{L_{\text{N}} } \mathord{\left/ {\vphantom {{L_{\text{N}} } L}} \right. \kern-0pt} L}$ to express resource dependence.

### Natural resource dependence and its crowding-out effect on human capital

Natural resource dependence could be borne out of the large-scale exploitation of natural resources or the rising prices of natural resource products. This induces the crowding-out effect on human capital and physical capital thereby restraining technological progress. As a result, the dependence on natural resources results in a lack of human capital and innovation and leads to deterioration in endowment, which is not conducive to economic growth. Since most of these natural resources are non-renewable, the use of unskilled human capital and primitive technology deteriorates them further and slows down economic growth. Thus, the resource curse phenomenon occurs.

When the price of a natural resource product $P_{\text{N}}$ increases, the natural resource sector increases the demand for labor, thus changing the factor income proportion of the natural resource sector to that of the manufacturing sector. In the absence of factor mobility barriers between the two departments, manufacturing employees will flow to the natural resource sector. It is generally believed that the natural resource sector is a low-demand sector for human capital and that the development of manufacturing requires more human capital. Therefore, a loss in manufacturing jobs leads to a decline in the human capital stock of manufacturing $H_{\text{M}}$ and further leads to an actual decline in the use of human capital in the whole economy. At the same time, the deepening of dependence on natural resources leads to a reduction in the demand for human capital, resulting in a decline in the rate of return on human capital investment that affects the formation of human capital. As a result, the level of per capita human capital is low.

In this context, how is it possible to avoid the crowding-out effect of natural resource dependence on human capital? Although many factors affect the formation of human capital (e.g., investment in health and medical facilities and entrepreneurial training programs), education is the main contributor to human capital accumulation. Natural resource dependence decreases the willingness of individuals to invest in human capital. However, the government can increase public expenditures for education and improve the private rate of return on human capital investment, thereby improving the level of human capital.

Natural resource dependence has a crowding-out effect on human capital, but public investment in education contributes to the accumulation of human capital. While dependence on natural resource inhibits human capital development, public investment in education might promote human capital accumulation. Such an increase in public education investment can reduce the crowding-out effect of natural resource dependence on human capital.

## Empirical analysis

### Empirical research design

The panel data from 31 provinces in China collected from 1999 to 2015 is used in the empirical analysis. The data used in the regressions is from the CSMAR database, “China Compendium of Statistics (1949–2008)” and “Statistical Communique of the Provinces on the National Economic and Social Development”.

First, we estimate the effect of natural resource dependence on human capital with the following model:

5 $$H_{it} = \beta_{0} + \beta_{1} D_{it} + \beta_{2} E_{it} + \beta_{3} U_{it} + \beta_{4} G_{it} + \varepsilon_{it}$$

where *i* is a provincial index; *t* indicates time; *H* represents the accumulation of human capital and can be measured by the number of college students divided by the total population; *D* is the variable of natural resource dependence and can be measured by dividing the number of individuals employed by the mining sector by the number of employees in urban areas; and *E* indicates public education investment and can be measured by dividing public education expenditures by the gross domestic product (GDP). We also introduce urbanization and economic growth rate as control variables. *U* represents the level of urbanization and can be measured by dividing the urban population by the total population; *G* is the growth rate of the gross regional domestic product; and $\varepsilon$ represents the error term.

Then, we include the interaction term between natural resource dependence and public education investment in the regression. This interaction term enables us to test whether the negative effect of natural resource dependence on human capital accumulation decreases with public education investment, with the following regression equation:

6 $$H_{it} = \beta_{0} + \beta_{1} D_{it} + \beta_{2} E_{it} + \beta_{3} U_{it} + \beta_{4} G_{it} + \beta_{5} D_{it} \times E_{it} + \varepsilon_{it}$$

There are major differences in the economic development of various regions in China. The economic development level in eastern regions is higher than that in the central and western regions. This situation raises questions about whether these differences affect relationships between natural resource dependence, public education investment, and human capital accumulation. To explore this issue, we analyze the data according to region, separating data from the eastern region from data from the central and western regions. The eastern region includes Beijing, Tianjin, Shanghai, Liaoning, Hebei, Shandong, Jiangsu, Zhejiang, Fujian, Guangdong, and Hainan; the central and western regions include Shanxi, Inner Mongolia, Jilin, Heilongjiang, Anhui, Jiangxi, Henan, Hubei, Hunan, Guangxi, Chongqing, Sichuan, Guizhou, Yunnan, Tibet, Shaanxi, Gansu, Qinghai, Ningxia, and Xinjiang. The descriptive statistics based on these data are presented in Table 1.

**Table 1:** Descriptive statistics

	Whole sample (1)		Eastern region (2)		Central and western regions (3)		Statistical significance of mean difference between (2) and (3) *P* values
	Mean	Variance	Mean	Variance	Mean	Variance	
*H*	0.013	0.000	0.017	0.000	0.011	0.000	< 0.001
*D*	0.031	0.001	0.016	0.0003	0.039	0.0009	< 0.001
*E*	0.032	0.000	0.023	0.000	0.037	0.000	< 0.001
*U*	0.476	0.026	0.606	0.029	0.404	0.01	< 0.001
*G*	0.11	0.001	0.112	0.006	0.113	0.007	0.804
Observations	527		187		340		

*P* values were obtained in a two-tailed test

As shown in Table 1, the human capital level in the eastern region is significantly higher than that in the central and western regions; the natural resource dependence of the central and western regions is much higher than that of the eastern regions; the public education investments of the central and western regions are significantly higher than that of the eastern region; the urbanization level in the eastern region is much higher than that in the central and western regions; and there are no significant differences in the economic growth rates of the eastern region and the central and western regions.

### Regression results: total sample

Before the panel data model is applied, it is necessary to determine which model should be adopted: the random effect model or fixed effect model. We employed the Hausman test to determine the appropriate model. The results show that the hypothesis that there is no systematic difference between the fixed effect model and the random effect model should be rejected, and that the fixed effect model is superior. The estimated results of the fixed effect model are shown in Table 2.

**Table 2:** Effect of natural resource dependence on human capital: regression results for total sample

Explanatory variable	Model 1		Model 2		Model 3		Model 4	
	Coefficient	*P* value	Coefficient	*P* value	Coefficient	*P* value	Coefficient	*P* value
Constant	0.023	0.000	0.012	0.000	− 0.016	0.000	− 0.014	0.000
*D*	− 0.322	0.000	− 0.22	0.000	− 0.114	0.000	− 0.161	0.000
*E*			0.264	0.000	0.099	0.000	0.069	0.000
*U*					0.053	0.000	0.051	0.000
*G*					0.0004	0.000	0.0004	0.000
*D* × *E*							1.692	0.000
*R* ^2^	0.616		0.744		0.895		0.898	
*F* value	25.57		44.92		123.69		124.5	
Observations	527		527		527		527	

Model 1 in Table 2 is a single-variable regression model. Natural resource dependence is negatively correlated with human capital level. When natural resource dependence is increased by 0.01, the level of human capital is reduced by 0.003. Given the average human capital level, from the economic perspective, natural resource dependence has a major effect on the level of human capital. Model 2 in Table 2 introduces the public education investment variable so that the negative effect of natural resource dependence on the level of human capital is reduced, but it remains statistically significant. Public education investment has a significant positive effect on the level of human capital. Model 3 in Table 2 introduces urbanization level and economic growth rate as control variables. In Model 3, the negative effect of natural resource dependence on the level of human capital is further reduced, but it is still statistically significant. Moreover, the positive effect of public education investment on the level of human capital is also reduced but is still statistically significant.

As previously mentioned, the higher level of public education investment reduces the negative effect of natural resource dependence on human capital. Therefore, Model 4 in Table 2 includes the interaction term of natural resource dependence and public education investment. The regression results show that the coefficient of the interaction between natural resource dependence and public education investment is significantly positive. This result is consistent with our expectations. After inclusion of this interaction term, natural resource dependence retains its significant negative impact on the level of human capital, and public education investment retains its significant positive impact on the level of human capital.

Given the economic significance of the coefficient of the interaction term, it is important to investigate whether it is feasible to turn the negative effect of natural resource dependence on human capital into a positive effect. Therefore, the threshold of public education investment can be identified as follows:

7 $$\frac{{{\text{d}}H}}{{{\text{d}}D}} = \beta_{1} + \beta_{5} E \ge 0 .$$

According to Model 4 in Table 2, this threshold is 9.5%, indicating that public education investment can offset the negative effect of natural resource dependence on human capital. Indeed, the offset effect of public education investment increases as a function of public education investment. According to our results, if public education investment were to account for more than 9.5% of the GDP, this investment could not only offset, partially, the negative impact of natural resource dependence on human capital, but also turn the negative effect into a positive one. In other words, if public education investment level were high enough, the higher degree of natural resource dependence could actually increase the level of human capital.

### Subsample regression results

Separate analyses of the data from the eastern region and the central and western regions according to Eqs. (5) and (6) show that the negative effect of natural resource dependence on human capital is statistically significant only in the central and western regions. From the economic perspective, the crowding-out effect of natural resource dependence on human capital in the central and western regions was much more significant than that in the eastern regions. From a broader perspective, the crowding-out effect of natural resource dependence on human capital existed only in the central and western regions.

Model 4 in Table 3 allows calculation of the threshold value of public education investment, which enables natural resource dependence to have a positive effect on human capital accumulation. In the central and western regions, natural resource dependence would have a positive impact on human capital accumulation if public education investment accounted for 6.97% of the GDP, but the actual investment in public education in these regions accounted for only 3.7% of the GDP. However, the data show that public education investment has a direct positive effect on human capital accumulation. Moreover, by reducing the crowding-out effect of natural resource dependence on human capital, public education investment also has an indirect effect on human capital accumulation. Therefore, the central and western regions should gradually increase their investment in public education.

**Table 3:** Effects of natural resource dependence on human capital: sub sample regression results

Explanatory variable	Eastern region				Central and western regions			
	Model 1		Model 2		Model 3		Model 4	
	Coefficient	*P* value	Coefficient	*P* value	Coefficient	*P* value	Coefficient	*P* value
Constant	− 0.026	0.000	0.027	0.000	− 0.251	0.000	− 0.007	0.000
*D*	− 0.137	0.18	− 0.05	0.403	− 0.357	0.000	− 0.184	0.000
*E*	0.21	0.000	0.259	0.000	0.101	0.000	0.052	0.000
*U*	0.056	0.000	0.059	0.000	0.046	0.000	0.04	0.000
*G*	0.0006	0.000	0.0006	0.000	− 0.0003	0.000	0.0004	0.000
*D* × *E*			− 10.413	0.51			2.638	0.000
*R* ^2^	0.922		0.927		0.862		0.88	
*F* value	145.85		145.17		86.08		96.44	
Obs.	187		187		340		340	

## Conclusions

Our analysis of the relationships between natural resource dependence, public education investment, and human capital accumulation based on panel data from 31 provinces in China collected from 1999 to 2015 reveals a significant negative correlation between natural resource dependence and human capital accumulation, suggesting a crowding-out effect of natural resource dependence on human capital. The results are consistent with our theoretical expectations. Based on our theoretical prediction, we also discuss the relationship between natural resource dependence and human capital accumulation by introducing the interactive term of natural resource dependence and public education investment. The results show that, after the introduction of the interaction term, the coefficient of natural resource dependence remains significantly negative, but the interactive coefficient is significantly positive. The results indicate that public education investment could reduce the crowding-out effect of natural resource dependence on human capital.

Our regional analysis shows that the natural resource dependence in the central and western regions is much higher than in the eastern regions. Moreover, the crowding-out effect of natural resource dependence on human capital operates only in the central and western regions. Indeed, public education investment could reduce the crowding-out effect of natural resource dependence on human capital in the central and western regions.

Endogenous growth theory emphasizes the role of human capital in long-term economic growth. The crowding-out effect of natural resource dependence on human capital inevitably leads to a low economic growth rate in the long term and a low level of economic development, resulting in the phenomenon of the resource curse. The crowding-out effect of natural resource dependence on human capital is among the mechanisms by which the resource curse influences economies, raising questions about how to reduce or even avoid the crowding-out effect of natural resource dependence on human capital. According to our results, it is necessary to increase public investment in education in regions with higher natural resource dependence. The government should utilize the income of the natural resource sector to increase education investment to enhance the human capital of residents. In particular, measures can be taken from two aspects: demand and supply.

First, from the perspective of demand, the demand for human capital is from the enterprises. Because the level of human capital in the industrial sector is higher than that in the agricultural sector, the government should support the development of industrial sectors in natural resources-rich areas. Only when the industrial sectors in these areas develop, the demand for human capital increases, and the gains from human capital increase, do residents have the desire to invest more in human capital. In this way, the development of the industrial sector will bring about a general improvement in the level of human capital.

Second, from the point of view of supply, education is one of the main ways to accumulate human capital. The government should set up resource reserve funds to increase investment in education and encourage social capital to invest in education. This will reduce the cost of human capital investment by the individual. In addition to strengthening education investment, we can also strengthen the accumulation of human capital by the way of talent hunt; give subsidies and preferential policies to the talents in the region in terms of wages, research funds and living conditions, which can attract scarce talents in the short term.

This paper only discussed the transmission mechanism of the crowding-out effect of natural resource dependence on human capital. Actually the transmission mechanism in the real economy is more complex, and often multiple transmission mechanisms work together, so how can the size of each mechanism be measured? Is there a more general factor behind the multiple mechanisms? These are questions that need to be further studied.
